# A case report of Multiple Symmetric Lipomatosis (MSL) in an East Asian Female

**DOI:** 10.1186/s12905-020-01055-w

**Published:** 2020-09-14

**Authors:** Kyunghun Jung, Soonchul Lee

**Affiliations:** Department of Orthopaedic Surgery, CHA Bundang Medical Center, CHA University School of Medicine, 335 Pangyo-ro, Bundang-gu, Gyeonggi-do Republic of Korea

**Keywords:** Lipoma, Multiple, Symmetric, Female, Alcohol

## Abstract

**Background:**

Multiple Symmetric Lipomatosis (MSL) is a rare disorder related to fat metabolism and lipid storage. The condition results in characteristic depositions of fat, especially around the cephalic, cervical, and upper thoracic subcutaneous. It is much more common in adult males who live in the Mediterranean region and has only rarely been reported in Asian females. In this report, we present a case of an Asian female with MSL and also review the clinical features of the condition, including radiological and histological findings required for proper diagnosis and management.

**Case presentation:**

A 59-year-old Korean female came in with a chief complaint of palpable mass present in shoulder and upper back regions. Images showed diffuse non-encapsulated adipose tissue in the subcutaneous layer of the suboccipital, posterior neck area. The patient wanted to remove the mass for cosmetic reasons and discomfort. Excisional biopsy was planned. Preoperative blood analyses showed deteriorated liver function, and the computed tomography findings were consistent with liver cirrhosis. Detailed history taking revealed that she consumed highly levels of alcohol. Lipectomy was performed and the histological findings demonstrated large dystrophic adipocyte morphology. The patient was recovered uneventfully.

**Conclusion:**

When patients have multiple symmetric lipomatous lesions, clinicians should suspect MSL and survey possible associated conditions, such as alcoholism, liver cirrhosis, dyspnea, and neuropathy in detail.

## Background

Multiple Symmetric Lipomatosis (MSL), also known as Madelung’s disease, is a rare disorder related to fat metabolism and lipid storage. The condition results in characteristic depositions of fat, especially around the cephalic, cervical, and upper thoracic subcutaneous regions [1, 2]. After the first case reported by Benjamin Brodie in 1846, the incidence of MSL was found to be 1 in 25,000 [3]. Also known as Launois-Bensaude syndrome [4], MSL can be classified into four discrete phenotypes according to anatomic distributions of adipose tissue. MSL type 1 patients typically exhibit pseudo-athletic appearances with symmetrical distribution of fat mainly in the upper body. MSL type 2 patients appear obese due to the presence of pathological adipose tissue. MSL type 3 (i.e., the gynecoid type) affects the lower body areas, especially in thighs and medial sides of the knees, whilst MSL type 4 affects the abdominal area [5].

Although the exact mechanism of MSL is unknown, the condition is thought to be closely related to alcohol abuse. In this context, it is frequently reported in male in their 40’s to 70’s, and affects males up to 30 times more frequent than females [6]. Due to unknown reasons, cases of the disease have been more frequently reported in specific geographical areas (e.g., the Mediterranean region) [4, 7]. However, the condition has only rarely been reported in Asian females [8, 9]. In this report, we present a case of an Asian female with MSL. We also review the clinical features of the condition, including radiological and histological findings required for proper diagnosis and management.

## Case presentation

**Fig. 1:**
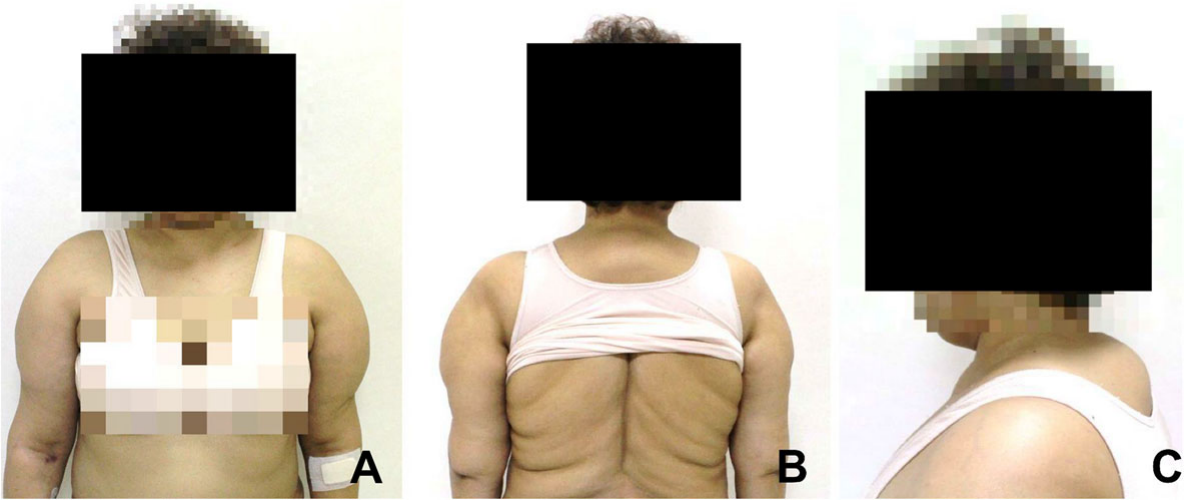
Preoperative distribution of masses. There was symmetrical swelling caused by masses of adipose tissue in both shoulders, the back, and the posterior neck, but the anterior neck was relatively spared. Anterior view (**a**), posterior view (**b**), neck lateral view (**c**)

**Fig. 2:**
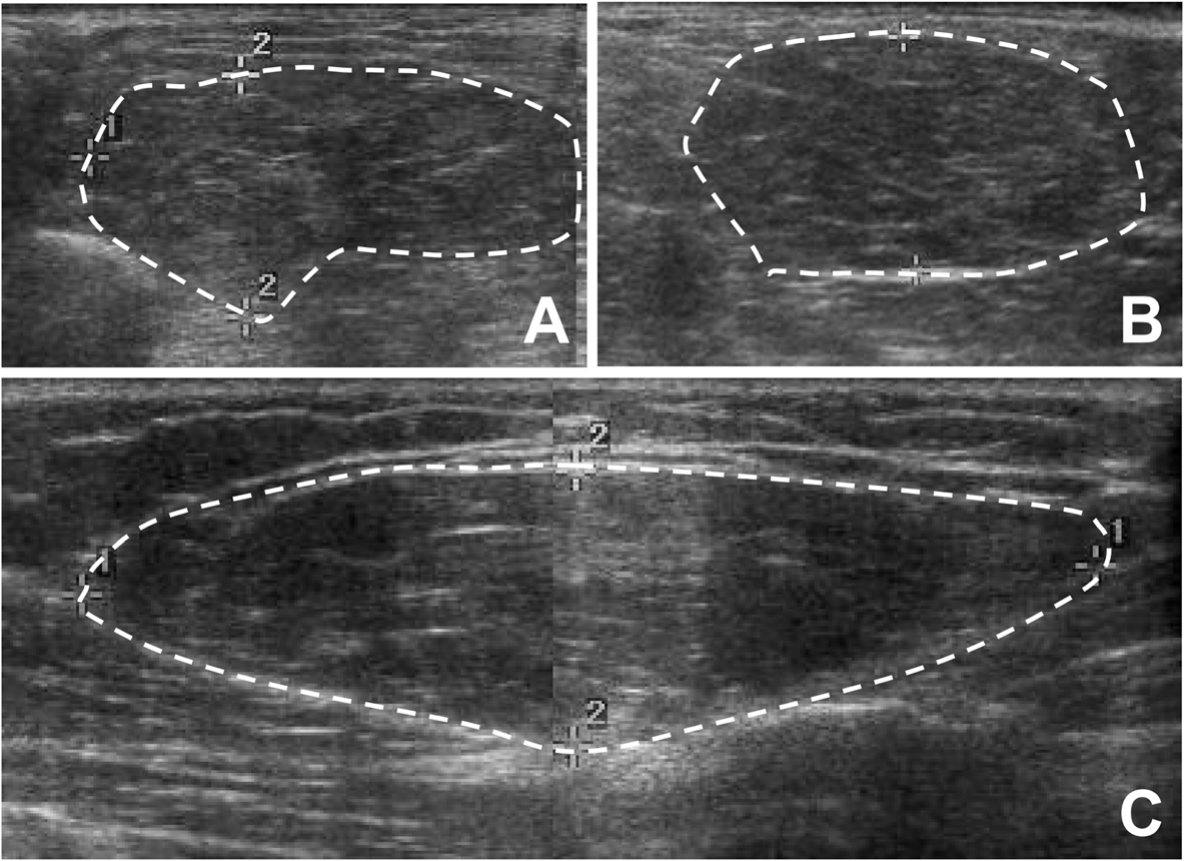
Ultrasonography findings. Ultrasonography indicated excessive adipose tissue (white lines) located in the subcutaneous layer of the shoulders (**a**, 5.73 × 2.12 × 4.32 cm), posterior neck (**b**, 4.7 × 7.7 × 2.0 cm), and back (**c**, 8.2 × 8.6 × 2.2 cm). All masses were compressible and had minimal vascularity

**Fig. 3:**
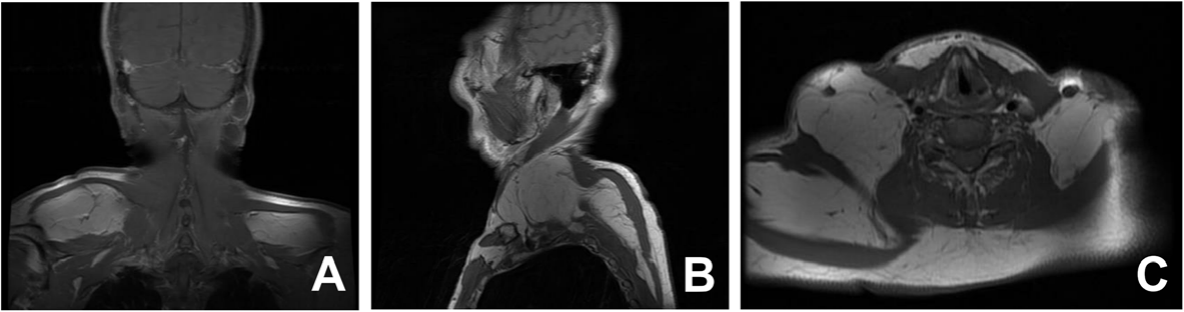
Magnetic resonance images of the patient’s neck. Coronal and sagittal T1 weighted sequences from the neck MRI revealed subcutaneous adipose tissue in both the shoulders (**a**) and posterior neck (**b**). Axial T1 weighted sequence findings also revealed diffuse subcutaneous fatty infiltration in both shoulders, but there was no mediastinal lipomatosis (**c**). MRI: Magnetic resonance image

**Fig. 4:**
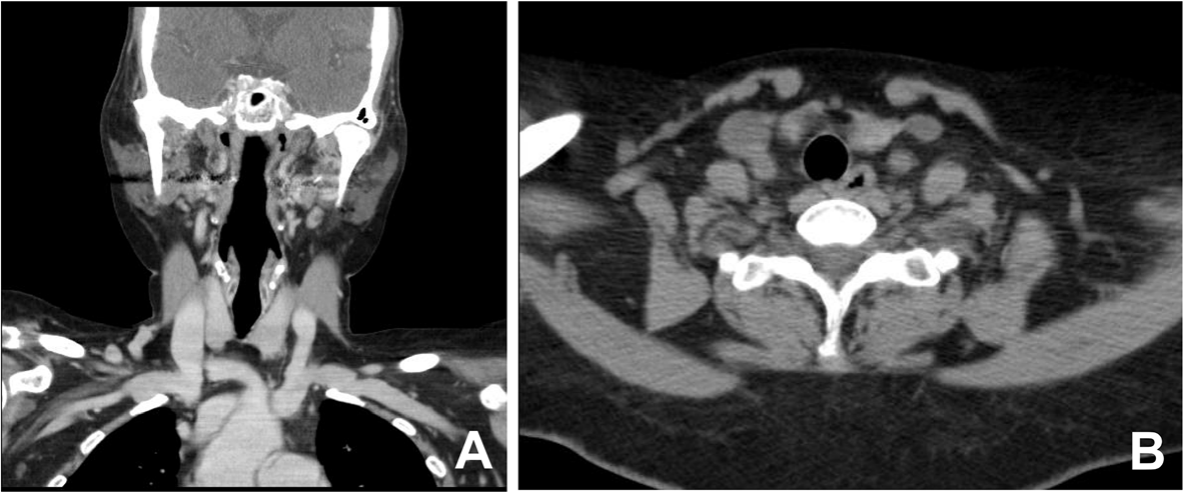
Computed tomography image of the patient’s neck. CT images were taken to evaluate the trachea and esophagus. There was no apparent airway or esophageal obstruction on the coronal view (**a**) or the axial view (**b**). CT: Computed tomography

**Fig. 5:**
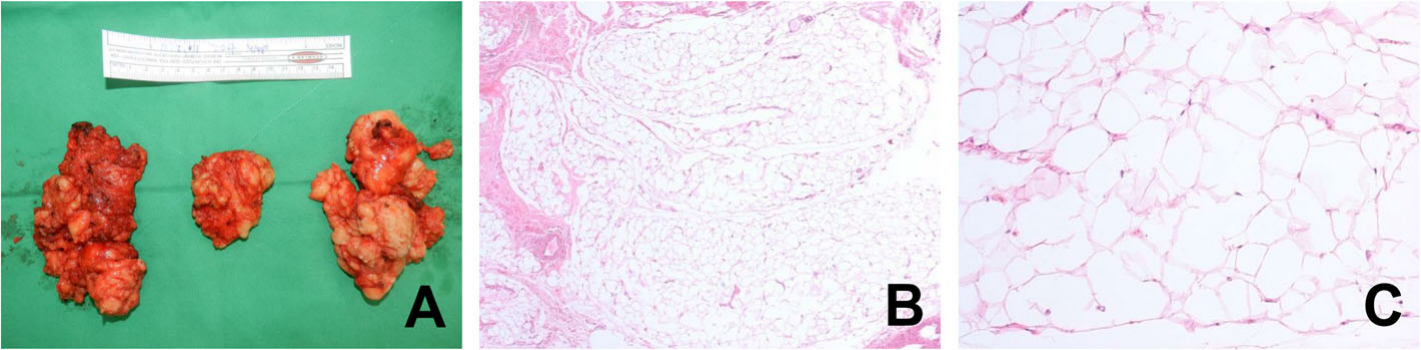
Gross photo and histologic finding of the excised mass. Specimens consisted of well-defined, pale yellow, fatty tissue. They had homogeneous, soft, solid, greasy cut surfaces without hemorrhage or necrosis. They were 8 × 4.5 × 2 cm (left), 8 × 4 × 2 cm (middle), and 6 × 4.5 × 1.5 cm (right) (**a**). Hematoxylin and eosin staining of the biopsy sample showed fatty tissue without any signs of malignant transformation. The adipocytes had significantly larger cross-sectional surface areas of the lipid vacuoles, compared with those from normal regions (40× (**b**), 200× (**c**)). The estimated weights were 64.8 g (Left), 57.6 g (Middle), 40.5 g (Right)

A 59-year-old Korean female came in with a chief complaint of palpable mass present in shoulder and upper back regions (Fig. 1). She had not previously received any diagnosis of disease except for psychiatric conditions. Height, weight, and BMI of the patient were 158 cm, 63 kg, and 25.2, respectively. Physical examination was done, and other than the presence of palpable protruding mass, no clinical signs or specific symptoms (e.g., pain or numbness) have been found. Ultrasonography was the first imaging study performed (Fig. 2), which revealed characteristics of lipoma. Computed tomography (CT) images and magnetic resonance images (MRI) were obtained to evaluate the depth and distribution of mass. Diffuse non-encapsulated adipose tissues were found in subcutaneous layers of suboccipital and posterior neck. There was no mass surrounding the tracheal area (Figs. 3, 4). Partial excision of the mass was planned because the patient wanted it removed for cosmetic reasons and physical discomfort. There were no apparent brain lesions in MRI and no specific findings in neurologic examinations. The preoperative evaluation included plain radiography of the thorax, electrocardiography, and blood analyses. Our patient had impaired fasting glucose levels and elevated aspartate aminotransferase, alanine aminotransferase, and uric acid levels. The values for total cholesterol and rheumatic factor, anti-cyclic citrullinated peptides, and antinuclear antibody levels were within the reference ranges. The laboratory findings are presented in Table 1. Homeostatic Model Assessment for Insulin Resistance (HOMA-IR) and Homeostatic Model Assessment for beta-cell function (HOMA-B) scores were 2.6 and 68.7% respectively, which meant that the patient had early insulin resistance. Additional history taking revealed that she had a history of high alcohol consumption. The patient consumed 48.6 g of alcohol daily, which is a high risk for alcohol consumption according to WHO criteria [10, 11].. Abdominal ultrasound and liver CT were performed in order to determine any existing hepatobiliary problems. Mild gallbladder edema was found in ultrasound. The CT scan found liver cirrhosis with mild splenomegaly. Consequently, the patient received a diagnosis of compensated alcoholic liver cirrhosis (Child-Turcotte-Pugh Classification B) due to heavy alcohol consumption. Under general anesthesia, excisional biopsies were performed via lipectomy at accumulated areas (Fig. 5a). The histological findings revealed large dystrophic adipocyte morphology. However, there were no findings of any sarcomatous changes (Fig. 5b, c). There were no problems with wound healing (e.g., infection, hematoma, or seroma formation) during the post-operative period. There was no recurrence or obvious enlargement of the remnant mass during the 3 years of follow-up observation.

**Table 1:** Findings for hepato-renal function, lipid metabolism, and autoimmune disease parameters in a patient with multiple symmetric lipomatosis

	Parameter	Case	Normal range		
			Min.	Max.	Unit
Autoimmune disease	ANA	Negative			
	Anti-CCP	Negative			
	RF	7.32	0		
	ESR	3	1	10	mm/h
	CRP	0.08	0	0.3	mg/dL
Renal function	BUN	15.5	8	23	mg/dL
	Creatinine	1.3	0.6	1.2	mg/dL
Liver function	Glucose	178	70	110	mg/dL
	T.PRO	5.1	5.8	8	g/dL
	Albumin	2.4	3.1	5.2	g/dL
	r-GTP	104	0	40	IU/L
	D.Bil	1.76	0	0.3	mg/dL
	T.Bil	2.54	0	1.2	mg/dL
	PT (INR)	1.46	0.89	1.09	
	aPTT	41	26	38	%
	Platelet count	78 k	150 k	380 k	/μL
	AST (GOT)	85	10	40	IU/L
	ALT (GPT)	20	5	40	IU/L
	ALP	140	35	129	IU/L
Lipid metabolism	Total cholesterol	50	0	200	mg/dL
	HDL cholesterol	10.2	35	65	mg/dL
	LDL cholesterol	31	77	135	mg/dL
	Triglycerides	1029	50	200	mg/dL
Others	Calcium	8.5	8.5	10.5	mg/dL
	Phosphorus	1.6	2.5	5.5	mg/dL
	Uric acid	10	2.5	8.3	mg/dL

ANA: Antinuclear antibody, Anti-CCP: Anti-citrullinated peptides, RF: Rheumatic factor, ESR: Erythrocyte sedimentation rate, CRP: C-reactive protein, BUN: Blood urea nitrogen, T.PRO: Total protein, r-GTP: R-glutamyl transpeptidase, D.Bil: Direct bilirubin, T.Bil: Total bilirubin, PT: Prothrombin time, aPTT: Activated partial thromboplastin time, AST: Aspartate aminotransferase, ALT: Alanine aminotransferase, ALP: Alkaline phosphatase

## Discussion and conclusions

In this report, we have described a case of a middle-aged East Asian female with MSL. MSL is a rare adipocyte metabolic disorder [12], with only a few report published about the disease. Patients with MSL present features of symmetrical fat localization on their neck, shoulder, trunk, upper arms, and occiput [1, 3, 13–15]. MSL is often confused with simple obesity by its similar clinical features and symptoms, hence it is important to identify key hallmarks to differentiate the two conditions [9].

An association between alcohol overconsumption and the disease has also been reported. There is no clear mechanism for alcohol and localized fat accumulation. However, it is known that alcohol damages adrenergic lipolysis by affecting enzyme processes in mitochondria. It can be a cofactor inducing a change in the number and function of b-adrenergic receptors. Alcohol abuse is likely the cause of uncontrolled accumulation in adipose tissue [7, 12, 16–19].. Kimiskidis et al. reported 60–90% of patients as also being heavy consumers of alcohol [18, 20]. Although the patient in this case report had disorderly adipocyte differentiation, it is not yet clearly determined whether hypercholesterolemia, diabetes mellitus, thyroid dysfunction, kidney, or liver disease is the associated cause of this change [21, 22]. The exact metabolic mechanism has not been elucidated, but the adipocytes of MSL are different from normal cells in proliferation, hormonal regulation, and mitochondrial activity [23]. The hypothesis is that pathological adipocytes are linked to decreased ß-oxidation and lipolysis in mitochondria [24]. Based on this hypothesis, this disease is presumed to be a condition different from brown adipose tissue [12, 25]. The histological structure is dystrophic with characteristics of lipoma and liposarcoma [12]. The histopathological examination of the samples from this case revealed that adipocyte vacuoles have large dystrophic morphology, compared to normal adipocytes. However, sarcomatous change or development of MSL-associated liposarcoma have not yet been identified. Also, it was not clear whether all excised tissues were brown fat. It is hard to determine the metabolic benefit from brown fat, only through this single case report, but we found that the brown fat in the MSL is functionally defective. So, we think that this excised fat tissue is not beneficial to the patient [12].

Daniel et al. sub-divided MSL into three types. The morphologic features of this patient’s mass corresponded to type Ib, based on the presence of a mass in the posterior neck region and both shoulders [26] (Table 2). The direction in which adipose tissue spreads is not yet clear [27]. Frequently, lipoma distribution around neck occurs in patients with MSL. Depending on location, this condition is referred to as a horse collar of the cervical vertebra, a buffalo hump of the posterior neck, or as hamster cheek near both parotid areas [28]. The patient usually complains of a cosmetic deformity. Associated features include some combinations of motor, sensory, or autonomic neuropathies, and myopathy [29]. Careful assessments with CT and MRI findings should be performed to identify tumors around trachea or esophagus that are likely to cause dyspnea or dysphagia [28]. In this case, CT and MRI scans of multiple lipomas around neck and shoulder were performed; the mass did not compress trachea or esophagus. Our patient had symptoms of depression that were exacerbated due to body shape changes. However, it remains unclear whether the depression or mood changes were directly related to the MSL. No studies have found an association between psychiatric problems and MSL.

**Table 2:** Classification of multiple symmetric lipomatosis

Types		Affected body areas
Type I	Ia	Neck
	Ib	Neck, shoulder girdle, upper arms
	Ic	Neck, shoulder girdle, upper arms, chest, abdomen, upper and lower back
Type II		Hips, bottom, and upper legs
Type III		General distribution skipping head, forearms, and lower legs

Treatment is usually performed for cosmetic reasons or to alleviate symptoms such as dyspnea or dysphagia caused by mass-associated compression. Surgery can be performed to obtain symptomatic relief [16, 30], and it is the only effective treatment. Method includes lipectomy and liposuction [30]. Lipectomy is an effective treatment method because it involves complete removal of the mass and has a low risk of damage to peripheral vessels and nerves. Nonetheless, complications such as postoperative infection, hemorrhage, hematoma, and lymphatic fistula formation may arise from surgery. Liposuction is one of the most advantageous and widely-used cosmetic surgery methods. Compared to lipectomy, liposuction is easier to perform and is less invasive. However, the risk of recurrence is higher because it is difficult to completely remove lipoma [30]. Chen et al.’s systematic review found that both lipectomy and liposuction have advantages and disadvantages, but that lipectomy is performed in most cases [30]. Utmost care should be taken while administering anesthesia when masses are distributed around neck, which may increase the risk of airway obstruction [30]. Concurrent abstinence or reduction of alcohol intake is also considered necessary, but its effect on the regression of lipomatosis has not been determined [21].

Evidence that MSL is directly related to life expectancy is not clear, but a long term follow-up study showed incidences of somatic neuropathy and sudden death due to fat occupation in mediastinal space [31]. Motomu et al.’s 12-year follow-up study of one patient found no recurrence of mass, but the patient died of hemorrhagic shock due to hepatocellular carcinoma and hepatorenal syndrome [27]. Fonseca et al. reported the presence of neuropathy in about 85% of patients with MSL and an association with sudden cardiac death [17]. Because central nervous system involvement can occur, it is important to closely monitor for the presence of neurological symptoms and clinical signs [18].

In conclusion, a better understanding of the clinical features related to this rare disease may be important to identify the characteristics of an accurate diagnosis and effective management. The findings of possible associated conditions, such as alcoholism, liver cirrhosis, dyspnea and neuropathy, and the prevention of unnecessary surgery should be considered.

## Data Availability

The datasets used and/or analyzed during the current study are available from the corresponding author on reasonable request.

## Abbreviations

CT: Computed tomography images
MRI: Magnetic resonance images
